# Antagonizing Effects of Aspartic Acid against Ultraviolet A-Induced Downregulation of the Stemness of Human Adipose Tissue-Derived Mesenchymal Stem Cells

**DOI:** 10.1371/journal.pone.0124417

**Published:** 2015-04-24

**Authors:** Kwangseon Jung, Jae Youl Cho, Young-Jin Soh, Jienny Lee, Seoung Woo Shin, Sunghee Jang, Eunsun Jung, Min Hee Kim, Jongsung Lee

**Affiliations:** 1 Department of Convergence Biomedical Science & Engineering, Eulji University, Seongnam City, Gyunggi Do, Republic of Korea; 2 Skincure Life Science Institute, Seongnam City, Gyunggi Do, Republic of Korea; 3 Department of Genetic Engineering, Sungkyunkwan University, Suwon-Si, Gyeonggi-Do, Republic of Korea; 4 Viral Disease Division, Animal and Plant Quarantine Agency, 175 Anyang-Ro, Manan-Gu, Anyang-Si, Gyeonggi-Do, Republic of Korea; 5 Biospectrum Life Science Institute, Seongnam City, Gyunggi Do, Republic of Korea; 6 Department of Senior Healthcare, BK21 plus program, Graduated school, Eulji University, Seongnam City, Gyunggi Do, Republic of Korea; 7 Department of Physical Therapy, College of Health Science, Eulji University, Seongnam City, Gyunggi Do, Republic of Korea; 8 Department of Dermatological Health Management, College of Health Science, Eulji University, Seongnam City, Gyunggi Do, Korea; University of Thessaly, Faculty of Medicine, GREECE

## Abstract

Ultraviolet A (UVA) irradiation is responsible for a variety of changes in cell biology. The purpose of this study was to investigate effects of aspartic acid on UVA irradiation-induced damages in the stemness properties of human adipose tissue-derived mesenchymal stem cells (hAMSCs). Furthermore, we elucidated the UVA-antagonizing mechanisms of aspartic acid. The results of this study showed that aspartic acid attenuated the UVA-induced reduction of the proliferative potential and stemness of hAMSCs, as evidenced by increased proliferative activity in the 3-(4,5-dimethylthiazol-2-yl)-2,5-diphenyltetrazolium bromide (MTT) assay and upregulation of stemness-related genes OCT4, NANOG, and SOX2 in response to the aspartic acid treatment. UVA-induced reduction in the mRNA level of hypoxia-inducible factor (HIF)-1α was also significantly recovered by aspartic acid. In addition, the antagonizing effects of aspartic acid against the UVA effects were found to be mediated by reduced production of PGE2 through the inhibition of JNK and p42/44 MAPK. Taken together, these findings show that aspartic acid improves reduced stemness of hAMSCs induced by UVA and its effects are mediated by upregulation of HIF-1α via the inhibition of PGE2-cAMP signaling. In addition, aspartic acid may be used as an antagonizing agent to mitigate the effects of UVA.

## Introduction

Ultraviolet A (UVA) (320–380 nm) is believed to be deleterious or beneficial to cells and tissues [1–3]. Several reports suggest that UVA may affect the hypodermis where adipose tissue-derived mesenchymal stem cells, preadipocytes, and adipocytes exist [4–6]. In addition, the direct effects of UVA irradiation on adipose tissue-derived mesenchymal stem cells (hAMSCs), especially the stemness of hAMSCs, have recently been elucidated by our group [7]. In this report, UVA irradiation reduces the stemness of hAMSCs, and this effect is mostly due to the reduced expression of OCT4, NANOG, and SOX2, which are stemness genes. This reduced expression is suggested to be mediated by the activation of PGE_2_-cAMP-HIF-1α signaling [7].

OCT4, PIT-OCT-UNC (POU) transcription factor, plays a critical role in regulating the differentiation of embryonic stem (ES) cells and maintaining the pluripotent nature of the blastocyst inner cell mass [8]. OCT4 was shown to function in a complex with NANOG and SOX2 to activate and repress genes controlling stem cell identity and differentiation [8]. Hypoxia-inducible factors (HIFs) were also reported to affect the self-renewal and differentiation processes of stem cells by specific regulation of relevant genes and the key transcription factors involved in these processes [9–10].

To attenuate the negative effects of UVA irradiation on stemness, a cell-based compound library screen that was intentionally biased to select compounds with relatively low toxicity and high activity, was conducted. A HRE (Hypoxia Responsive Element)-luciferase reporter assay was used as the screening tool to evaluate the UVA-antagonizing effects of single compounds in hAMSCs. HRE-luciferase reporter activity is dependent on hypoxia-inducible factors (HIFs). Since UVA irradiation reduces HRE-luciferase reporter activity by reducing the mRNA level of hypoxia-inducible factor (HIF)-1α, we tried to obtain molecules which are able to attenuate UVA effects over 30%. During this screening, aspartic acid was selected as a candidate for use as a UVA-antagonizing agent. Aspartic acid at high concentrations is a toxin that causes hyperexcitability of neurons and is also a precursor of other excitatory amino acid—glutamates. Their excess in quantity and lack of astrocytic uptake induces excitotoxicity and leads to the degeneration of astrocytes and neurons [11]. Aspartate may also be employed as a neuropeptide-like co-transmitter by pathways that release either glutamate or GABA as their principal transmitter. Possible neurobiological functions of aspartate in immature neurons include activation of cAMP-dependent gene transcription and in mature neurons inhibition of CREB function, reduced BDNF expression, and induction of excitotoxic neuronal death [12]. However, there are no reports regarding other properties.

In this study, we found that aspartic acid antagonized the effects of UVA on stemness by reducing the production of PGE_2_ via inhibition of JNK and p42/44 MAPK.

## Materials and Methods

### Human adipose tissue-derived stem cell culture

Three kinds of hAMSCs were purchased from Invitrogen (Carlsbad, CA, USA), ATCC (Manassas, VA, USA), and Thermo Fisher Scientific, Inc. (Waltham, MA, USA), respectively. The cryopreserved cells were thawed at 37°C and then immediately cultured in MesenPRO RS^TM^ medium (Gibco, Carlsbad, CA, USA). The cells were then expanded using MesenPRO RS^TM^ medium to 5 passages. The medium was changed every three days until the cells were 70% confluent, at which time they were passaged.

### UVA irradiation

When the cells were 70% confluent, the medium was removed, and the cells were washed with PBS and gently overlaid with Dulbecco’s modified Eagle’s medium (DMEM) devoid of phenol red (Santa Cruz Biotechnology, Inc., Santa Cruz, CA, USA). The cells were then irradiated for between 21 sec to 36 min and 0.05 to 5 J/ cm^2^, which was not cytotoxic. The cells were irradiated by UVA in DMEM devoid of phenol red using a Vilber-Lourmat UVA table centered on 365 nm (TF-20L) at 25°C, which was controlled during the irradiation. In addition, during irradiation, the lid of the dish was opened to minimize UVA absorption by the plastic materials. A piece of glass with a thickness of 4-mm was placed above the table to absorb the residual UVB radiation.

### Cell proliferation

hAMSCs were irradiated with the indicated doses of UVA and then incubated with 1 to 100 μM of aspartic acid (Sigma—Aldrich, St. Louis, MO, USA) for three days under serum-free conditions (in DMEM devoid of serum, at 37°C in 5% CO_2_). Serum-free conditions were chosen to exclude unknown effects of exogenous serum, which can have compositions that vary based on the donor species, the age of the animal the serum was obtained from, its feedstock, and season. After three days, the cell proliferation was evaluated using the MTT assay, which is based on the conversion of a substrate containing a tetrazolium ring into blue formazan by mitochondrial dehydrogenases [13]. The level of blue formazan was then measured spectrophotometrically at a wavelength of 570 nm and used as an indirect index of cell proliferation. In addition, cell proliferation was measured using BrdU incorporation assay [14]. Detection of BrdU incorporation was performed by ELISA (BrdU Cell Proliferation Assay Kit, Cell Signaling Technology, Danvers, MA, USA) according to the manufacturer’s instructions.

### Assessment of the percentage of apoptotic cells and necrotic cells

To detect apoptotic and necrotic cells, Apoptotic/Necrotic/Healthy Cells Detection Kit was used (PromoCell GmbH, Heidelberg, Germany). Cells were stained with Hoechst 33342, FITC-Annexin V, and Ethidium Homodimer. This assay was conducted after the incubation of hAMSCs under the conditions described above. After washing with 1X binding buffer, the cells were observed under a fluorescence microscope (Zeiss Axiophoto 2, Carl Zeiss, Germany). In this assay, while apoptotic cells were stained green and blue, necrotic cells were stained red and blue. A minimum of 500 cells were scored from each sample.

### Small interference RNA (siRNA) and expression plasmid for HIF-1α

ON-TARGETplus SMARTpool human siRNAs against HIF-1α (L-004018-00-0020), HIF-2α (L-004814-00-0020), and ON-TARGETplus non-targeting siRNA (D-001810-10-05) were synthesized by Thermo Fisher Scientific, Inc. (Waltham, MA, USA). Cells were then transfected with the indicated siRNAs at 50 nM for 48 h using DharmaFECT transfection agent (Dharmacon Research, CO, USA) according to the manufacturer’s instructions.

### RNA preparation

Total cellular RNA was extracted from hAMSCs grown in serum-free culture medium with or without the indicated concentrations of aspartic acid in the presence or absence of the indicated doses of UVA irradiation for three days using TRIzol reagent (Invitrogen, Carlsbad, CA, USA) and purified with a RNeasy Mini Kit (Qiagen, CA, USA) according to the manufacturer’s instructions. All samples were DNase treated (Ambion, CA, USA) and subsequently analyzed on an Agilent Bioanalyser (Agilent Technologies, Waldbronn, Germany) and a NanoDrop 8000 Spectrophotometer (Thermo Scientific, Schwerte, Germany) to determine RNA concentration, purity, and integrity. Samples with an appropriate RNA integrity number (RIN, ≥8.0) and RNA purity (A_260_/A_280_ = 1.8~2.0) were used.

### cDNA synthesis

Purified RNA (1 μg) was reverse-transcribed in a 20 μl reaction mixture using the RevertAid^TM^ First Strand cDNA Synthesis Kit Oligo dT Primers (Fermentas, Canada) on a BioRad PTC-200 DNA Engine thermal cycler (BioRad, Hercules, USA). Briefly, the RNA samples and oligo (dT) primers were mixed and denatured at 70°C for 10 min. The tubes were immediately placed on ice for at least 1 min. The transcription mixture and RNase inhibitor were added, and the mixture was incubated at 37°C for 5 min. The first-strand cDNA synthesis was initiated after the addition of M-mulv, and the reverse transcriptase reaction was performed at 42°C for 1 h. Finally, the enzymes were inactivated at 70°C for 10 min. The reactions were performed in triplicate to reduce any differences in the efficiency of the reverse transcription reaction. The cDNA was stored at -80°C and diluted 1:5 with RNase-free water for use as the template in the real-time PCR analysis.

### Real-time RT-PCR (TaqMan) analysis

Real-time RT-PCR analysis was conducted using an ABI7900HT machine (Applied Biosystems). All TaqMan RT-PCR reagents, including primers and probes, were purchased from Applied Biosystems. TaqMan analysis was conducted using predesigned and optimized Assays on Demand (Applied Biosystems). The following assays were used: HIF-1α (ID: Hs00936371_m1), HIF-2α (ID: Hs01026149_m1), OCT4 (ID: Hs03005111_g1), NANOG (ID: Hs02387400_g1), SOX2 (ID: Hs01053049_s1), REX1 (ID: Hs01938187_s1), GAPDH (ID: Hs00266705_g1). The reaction parameters were as follows: 2-min 50°C hold, 30-min 60°C hold, and 5-min 95°C hold, followed by 45 cycles of 20-s 94°C melting and 1-min 60°C annealing/extension. All measurements were performed in duplicate or triplicate and the results were analyzed using the ABI sequence detector software, version 2.0 (Applied Biosystems). Relative quantitation was conducted using GAPDH as a reference gene, which was validated using the NormFinder software (supporting information in S1 Fig and S1 Table). Since all assays used were optimized for PCR efficiency by the manufacturer, mRNA expression levels were estimated by the delta ct values.

### Luciferase reporter assay

To assay for AP-1, NF-κB, CRE, and HRE (hypoxia response element) promoter activities, hAMSCs were transfected with AP-1 (Stratagene, La Jolla, CA, USA), NF-κB (Stratagene, La Jolla, CA, USA), CRE-Luc (Stratagene, La Jolla, CA, USA), or HRE-Luc reporter (Addgene, MA, USA) along with 1 μg of the Renilla luciferase expression vector, which was driven by a thymidine kinase promoter (Promega, Madison, WI, USA) (internal standard), using the DharmFECT Duo transfection reagent (Thermo Fisher Scientific, Inc., Waltham, MA, USA) according to the manufacturer’s protocols. Twenty four hours later, the cells were cultured in MesenPRO RS^TM^ medium for 24 h and then irradiated with the indicated doses of UVA. Following UVA irradiation, the cells were immediately incubated with the indicated concentrations of aspartic acid (Sigma—Aldrich, St. Louis, MO, USA) for 24 h. The luciferase activities were then assayed using a Luciferase Assay System (Promega, Madison, WI, USA). The cells were harvested, lysed, and centrifuged. Next, the supernatants were assayed for luciferase activity using a Dual Luciferase Assay system (Promega, WI, USA) and a LB953 luminometer (Berthold, Germany). The activities were expressed as a ratio of the AP-1, NF-κB, CRE, or HRE-dependent firefly luciferase activity to the control thymidine kinase Renilla luciferase activity (% control). Results were confirmed by eight independent transfections.

### Immunoblotting

hAMSCs were irradiated with the indicated doses of UVA and then incubated with the indicated concentrations of aspartic acid for one hour or three days under serum-free conditions. The cells were washed twice with cold PBS (Sigma—Aldrich, St. Louis, MO, USA) and then lysed in 150 μl of sample buffer (100 mM Tris-HCl), pH 6.8, 10% glycerol, 4% sodium dodecyl sulfate (SDS), 1% bromophenol blue, 10% β-mercaptoethanol (All reagents were from Sigma—Aldrich, St. Louis, MO, USA). Next, the samples were resolved by sodium dodecyl sulfate-polyacrylamide gel electrophoresis (SDS-PAGE) and transferred to Immobilon-P PVDF membranes (Millipore Corporation, Bedford, MA, USA). The membranes were incubated overnight at 4°C with anti-HIF-1α antibody (Novus Biologicals, Beverly, MA, USA), anti-HIF-2α antibody (Novus Biologicals, Beverly, MA, USA), and anti-β-actin antibody (Sigma—Aldrich, St. Louis, MO, USA). The membranes were subsequently washed three times with Tris-buffered saline containing Tween-20 (TBST) (Sigma—Aldrich, St. Louis, MO, USA) probed with horseradish peroxidase-conjugated secondary antibody (Sigma—Aldrich, St. Louis, MO, USA), and developed using an ECL (enhanced chemiluminescence) Western blotting detection system (Amersham Biosciences).

### Mapk phosphorylation analysis

The levels of phospho-SAPK/JNK(Thr183/Tyr185), phospho-p38 MAPK (Thr180/Tyr182), JNK, and p38 MAPK were measured using a PathScan Inflammation Multi-Target Sandwich enzyme linked immunosorbent assay (ELISA) Kit (Cell Signaling Technology, Danvers, MA, USA) according to the manufacturer’s instructions. The levels of phospho-p42/44 MAPK (Thr202/Tyr204) and p42/44 MAPK expression were also determined using a PathScan Cell Growth Multi-Target Sandwich ELISA Kit (Cell Signaling Technology, Danvers, MA, USA) according to the manufacturer’s instructions.

### Elisa

hAMSCs were irradiated with the indicated doses of UVA and then incubated with the indicated concentrations of aspartic acid for three days under serum-free conditions. After three days, the concentrations of PGE_2_ or cAMP in the culture supernatant were measured using Enzyme-linked Immunosorbent Assay (ELISA) kits (ENZO Life Sciences International, Inc., PA, USA). Culture supernatants were added to 96 well plates. Alkaline phosphatase-conjugated PGE_2_ or cAMP and antibodies to PGE_2_ or cAMP were added to the sample wells. The samples were incubated at room temperature for 2 h and then the wells were washed and the p-nitrophenyl phosphate (pNpp) substrate solution was added. Finally, the samples were incubated at room temperature for 1 h and the absorbance was read according to the manufacturer’s instructions.

### Statistical analysis

All data are expressed as the means ± SD. Comparison between the control and the treated group was evaluated by one way analysis of variance followed with the Tukey’s multiple comparison test using GraphPad Prism (5.0) (GraphPad, La Jolla, CA, USA). Significance was considered at *P* values less than 0.05.

## Results

### Aspartic acid attenuates the effects of UVA irradiation on proliferative potential and self-renewal of hAMSCs

In our previous report, we demonstrated that UVA irradiation suppresses the stemness properties of hAMSCs and its inhibitory mechanisms involve upregulation of PGE_2_-cAMP-HIF-1α signaling through activation of AP-1 and NF-κB differentiation [7].

**Fig 1:**
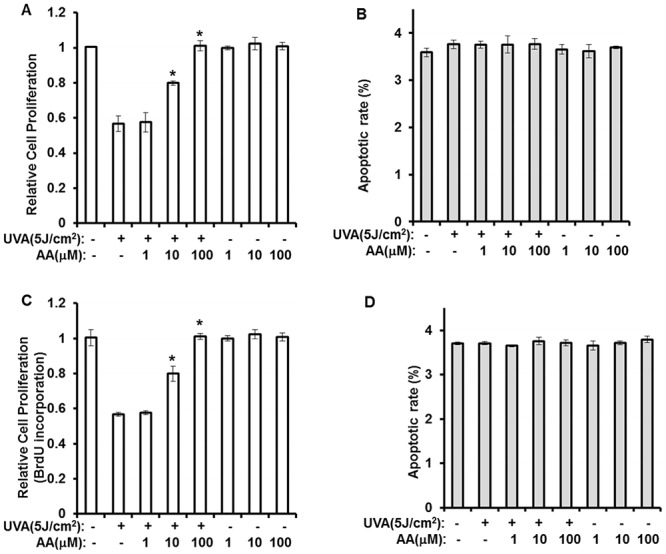
Aspartic acid attenuates the effects of UVA irradiation on proliferative potential of hAMSCs. hAMSCs were irradiated with 5 J/cm^2^ UVA and then incubated for three days in the presence of the indicated concentrations of aspartic acid under serum-free conditions. **(A)** After three days, cell proliferation was evaluated using the MTT assay **(A)** and BrdU incorporation assay **(C)**. The results were confirmed by five independent experiments, which were each conducted in duplicate. Data are expressed as the means ± S.D. *, *p*<0.05 vs. controls. **(B and D)** The apoptotic effects of UVA irradiation were determined by Hoechst 33332 staining. The results were verified by repeating the experiments four times, each of which was conducted in duplicate. Data are expressed as the means ± S.D. *, *p*<0.05 vs. controls.

To attenuate the negative effects of UVA on stemness, a cell-based compound library screen that was intentionally biased to select relative compounds with low toxicity and high activity was conducted. A HRE-luciferase reporter assay was used as the screening tool to evaluate the UVA-antagonizing effects of single compounds in hAMSCs. From this screen, aspartic acid was selected as a candidate for use as a UVA-antagonizing agent. As shown in Fig 1A and 1C, aspartic acid recovered UVA-induced reduction of proliferation in a dose dependent manner. Aspartic acid also did not show any apoptotic effects at the treated concentration (Fig 1B and 1D).

**Fig 2:**
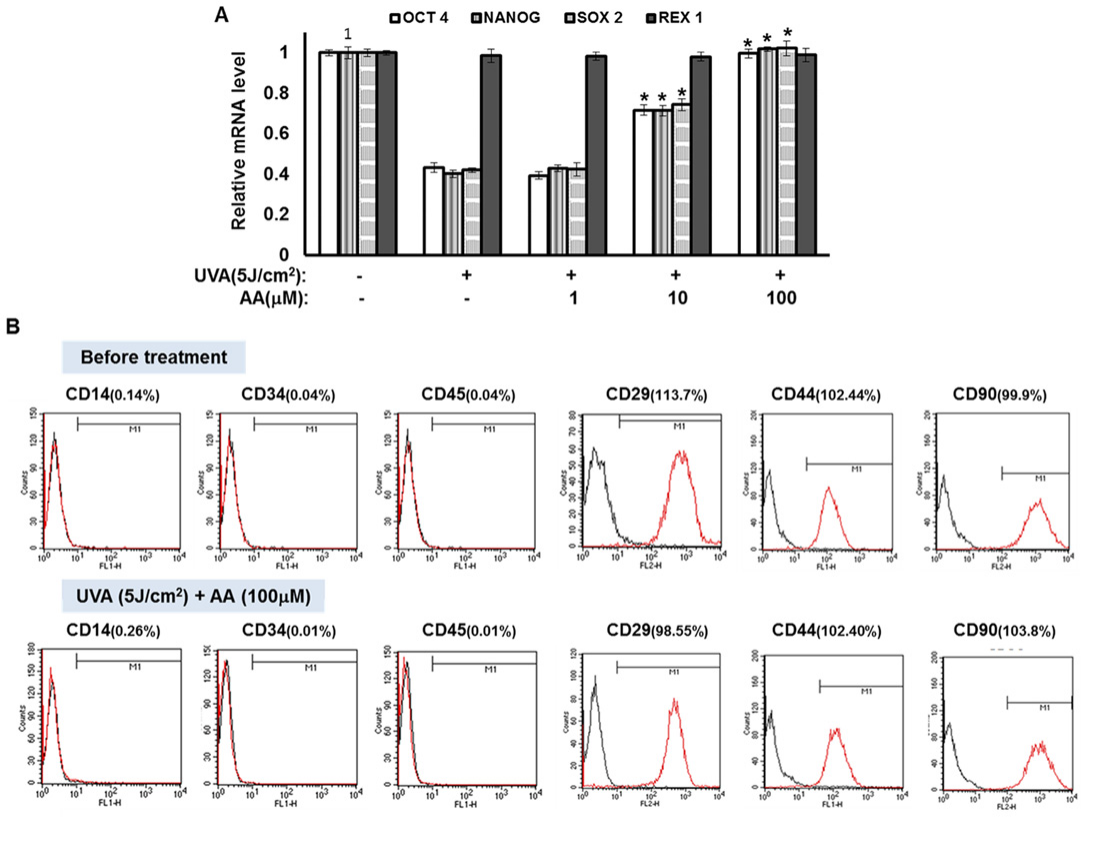
Aspartic acid attenuates the effects of UVA irradiation on self-renewal of hAMSCs. hAMSCs were irradiated with 5 J/cm^2^ UVA and then incubated for three days in the presence of the indicated concentrations of aspartic acid under serum-free conditions. **(A)** After three days of incubation under serum-free conditions, total RNA was isolated and the mRNA levels of the OCT4, NANOG, SOX2, and REX1 genes were measured by real-time quantitative RT-PCR. The results are expressed relative to untreated cells after normalization against the GAPDH. Data are expressed as the means ± S.D. *, *p*<0.05 vs. UVA (5J)-treated controls. The results were verified by repeating the experiments four times, each of which was conducted in duplicate. **(B)** Effects of UVA irradiation and aspartic acid treatment on expression pattern of cell surface markers were determined. AA: aspartic acid

In addition, as shown in Fig 2A, expression levels of OCT4, NANOG, and SOX2 which were reduced by UVA irradiation were all significantly recovered by aspartic acid. However, Rex 1 expression was not affected by aspartic acid and UVA irradiation. Moreover, we investigated the effect of the UVA irradiation and aspartic acid treatment on cell surface markers of hAMSCs, which include CD14, CD34, and CD45 as negative markers and CD29, CD44, and CD90 as positive markers. As shown in Fig 2B, the UVA irradiation and aspartic acid treatment changed the portion of CD29^+^, CD44^+^, and CD90^+^ cells. However, the extent of changed expression was not high. These results indicate that hAMSCs have adipose-derived stem cell surface markers and the expression pattern of these stem cell surface markers was not affected by the UVA irradiation and aspartic acid treatment. These results suggest that aspartic acid attenuates the effects of UVA irradiation on hAMSCs.

### UVA irradiation-induced downregulation of HIF-1α is restored by aspartic acid

**Fig 3:**
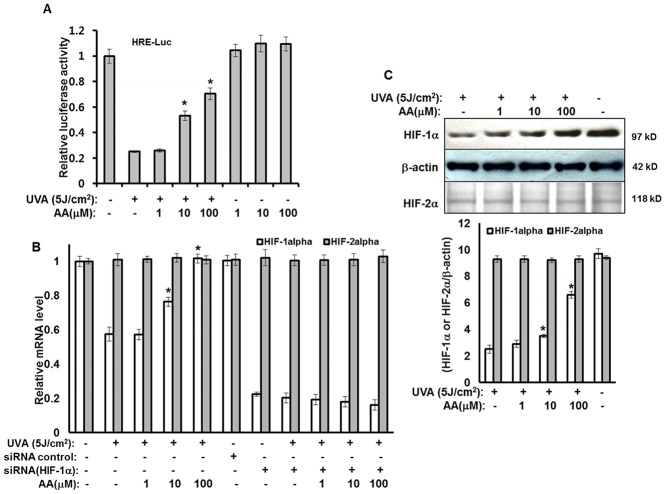
Aspartic acid increases downregulated expression of HIF-1α by UVA irradiation. **(A)** hAMSCs were transfected with HRE-Luc reporter along with a Renilla luciferase expression vector driven by a thymidine kinase promoter using DharmFECT Duo transfection reagent according to the manufacturer’s protocols. After incubation for 24 h, the cells were irradiated with 5J UVA and then further incubated in the presence of the indicated concentrations of aspartic acid under serum-free conditions for 14 h. These cells were then harvested, lysed, and assayed. The results were confirmed by three independent transfections. Data are expressed as the means ± S.D. *P<0.05 compared to the UVA (5J)-treated control. **(B)** hAMSCs were irradiated with 5 J/cm^2^ UVA or transfected with the siRNA for HIF-1α or 2α and then incubated for three days in the presence of the indicated concentrations of aspartic acid under serum-free conditions. After three days of incubation, total RNA was isolated and the mRNA levels of the HIF-1α and HIF-2α gene were measured by real-time quantitative RT-PCR. The results are expressed relative to untreated cells after normalization against GAPDH. Data are expressed as the means ± S.D. *, *p*<0.05 vs. UVA (5 J/cm^2^)-treated controls. The results were verified by repeating the experiments four times, each of which was conducted in duplicate. **(C)** Total lysates were analyzed by Western blot using the HIF-1α and HIF-2α antibodies. The results were verified by repeating the experiments three times. AA: aspartic acid

Our previous report demonstrated that expression of the HIF-1α gene plays an important role in the UVA-induced reduction of stemness [7]. To characterize the effects of aspartic acid on the reduction in expression of HIF-1α by UVA irradiation, HRE-luciferase reporter assay, real-time PCR, and Western analyses of HIF-1α and HIF-2α were conducted. In a luciferase reporter assay, UVA irradiation-induced reduction of HRE-luciferase reporter activity was found to be significantly recovered by aspartic acid treatment (Fig 3A). In addition, treatment with aspartic acid attenuated the reduction in the expression of HIF-1α that occurred in response to treatment with UVA irradiation (Fig 3B). However, aspartic acid did not affect the expression of HIF-2α (Fig 3B). Consistent with these findings, the reduction in the protein level of HIF-1α that was induced by UVA irradiation was also significantly recovered by aspartic acid (Fig 3C). Taken together, these findings suggest that aspartic acid recovered the reduced stemness of hAMSCs due to UVA irradiation through the upregulation of HIF-1α.

### Aspartic acid reduces UVA-induced production of PGE_2_ and cAMP through inhibition of JNK and P42/44 MAPK

**Fig 4:**
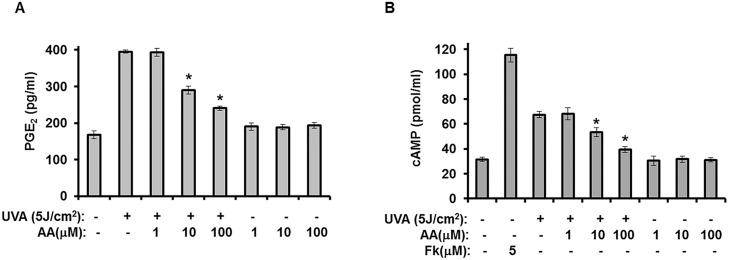
Aspartic acid reduces UVA-induced production of PGE_2_ and cAMP. hAMSCs were irradiated with 5 J/cm^2^ UVA and then incubated for three days in the presence of the indicated concentrations of aspartic acid under serum-free conditions. **(A and B)** After three days of incubation, the supernatants were harvested for PGE_2_ (A) and cAMP (B) measurement. Data are expressed as the means ± S.D. *, *p*<0.05 vs. UVA (5J)-treated controls. The results were verified by repeating the experiments three times, each of which was conducted in duplicate. AA: aspartic acid, Fk: forskolin.

Several papers have reported that the PGE_2_-cAMP signaling pathway downregulates the expression of HIFs [15,16]. In addition, UVA irradiation was found to downregulate HIF-1α expression through activation of the PGE_2_-cAMP signaling pathway [7]. The results of the present study indicate that UVA irradiation-induced reduction of HIF-1α was restored by aspartic acid. Therefore, we investigated the effects of aspartic acid on the increased production of PGE_2_ and cAMP caused by UVA irradiation. In this study, treatment of hAMSCs with aspartic acid led to a significant decrease in the production of PGE_2_ and cAMP, when compared to the UVA-irradiated controls (Fig 4A and 4B).

**Fig 5:**
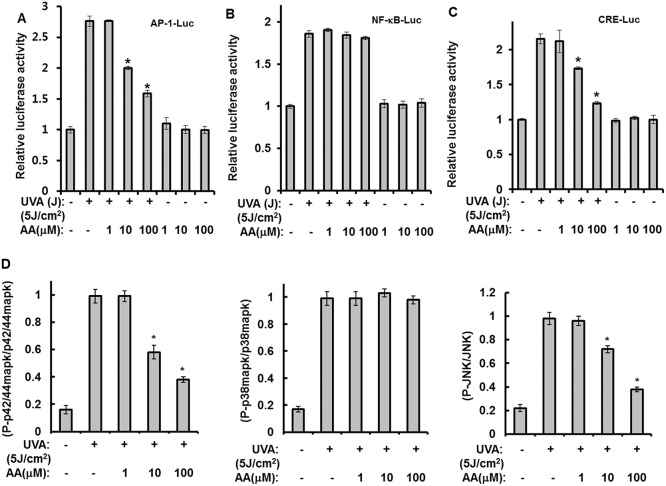
Aspartic acid reduces UVA-induced production of PGE_2_ and cAMP through the inhibition of JNK and p42/44 MAPK. **(A, B, and C)** hAMSCs were transfected with AP-Luc, NF-κB-Luc, or CRE-Luc reporters along with a Renilla luciferase expression vector driven by a thymidine kinase promoter using DharmFECT Duo transfection reagent according to the manufacturer’s protocols. After incubation for 24 h, the cells were irradiated with 5J UVA and then further incubated in the presence of the indicated concentrations of aspartic acid under serum-free conditions for 14 h. These cells were then harvested, lysed, and assayed. The results were confirmed by three independent transfections. Data are expressed as the means ± S.D. *P<0.05 compared to the UVA (5J)-treated control. **(D)** hAMSCs were irradiated with 5 J/cm^2^ UVA and then treated with the indicated concentrations of aspartic acid for 1 h under serum-free conditions. After 1 h of incubation, the cell lysates were analyzed using a Multi-Target Sandwich ELISA Kit. The results were verified by repeating the experiments three times, each of which was conducted in duplicate. AA: aspartic acid.

To investigate the action mechanisms of aspartic acid, luciferase reporter assays for AP-1, NF-κB, or CRE were conducted. As shown in Fig 5A, 5B, and 5C, while aspartic acid had no effect on the UVA-induced activation of the NF-κB promoter, activation of AP-1 and CRE by UVA irradiation was reduced. These results suggest that aspartic acid attenuated UVA-induced production of PGE_2_ through inhibition of AP-1 activity. Therefore, we further investigated the effects of aspartic acid on three types of MAPKs (JNK, p42/44 MAPK, and p38 MAPK). The levels of phospho-SAPK/JNK (Thr183/Tyr185), phospho-p38 MAPK (Thr180/Tyr182), JNK, and p38 MAPK were measured using a PathScan Inflammation Multi-Target Sandwich enzyme linked immunosorbent assay (ELISA) Kit (Cell Signaling Technology, Danvers, MA, USA). The levels of phospho-p42/44 MAPK (Thr202/Tyr204) and p42/44 MAPK expression were also determined using a PathScan Cell Growth Multi-Target Sandwich ELISA Kit (Cell Signaling Technology, Danvers, MA, USA).

Among the three types of MAPKs evaluated, the activities of JNK and p42/44 MAPK, but not p38 MAPK, were reduced by aspartic acid when compared to the UVA-irradiated controls (Fig 5D). Collectively, these results suggest that aspartic acid attenuates the UVA-induced reduction of stemness by reducing the production of PGE_2_ and cAMP via the inhibition of JNK and p42/44 MAPK.

### Antagonizing effects of aspartic acid against UVA-induced downregulation of stemness genes are mediated by downregulating PGE_2_-cAMP-HIF-1α signaling through inhibition of AP-1

**Fig 6:**
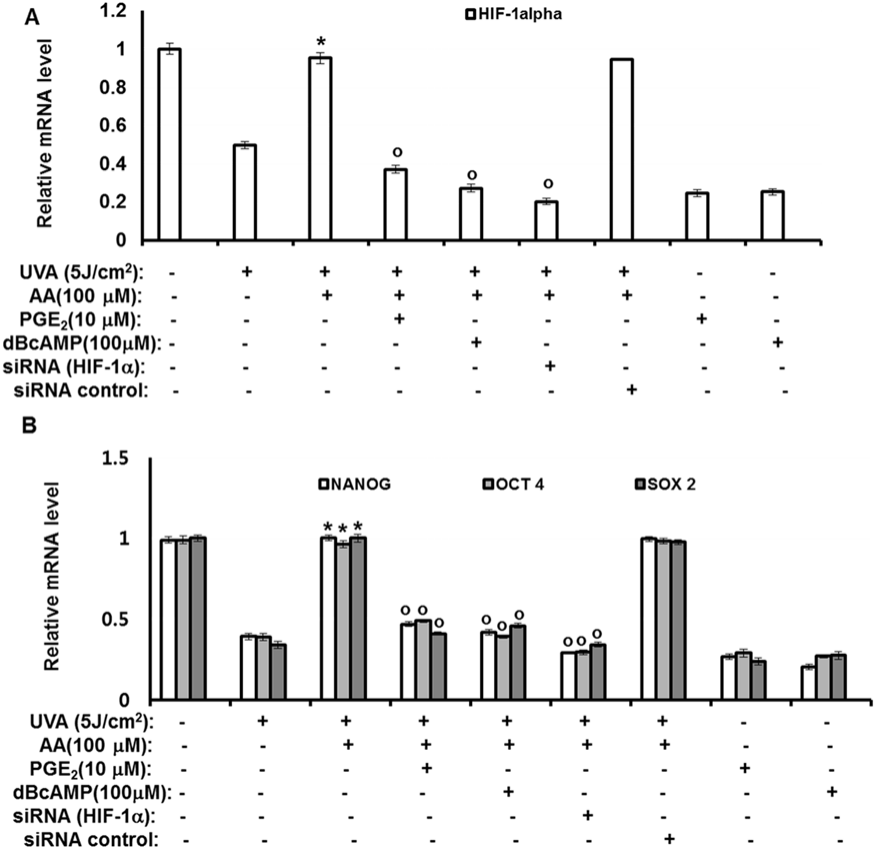
Antagonizing effects of aspartic acid against UVA-induced downregulation of stemness genes are mediated by downregulating PGE_2_-cAMP-HIF-1α signaling through inhibition of AP-1. hAMSCs were irradiated with 5 J/cm^2^ UVA or transfected with the siRNA for HIF-1α and then incubated for three days with aspartic acid (100 μM) in the presence of the indicated concentration of PGE_**2**_ or cAMP under serum-free conditions. After three days of incubation, total RNA was isolated and the mRNA levels of the HIF-1α gene (A) and OCT4, NANOG, SOX2 genes (B) were measured by real-time quantitative RT-PCR. The results are expressed relative to untreated cells after normalization against GAPDH. Data are expressed as the means ± S.D. *, *p*<0.05 vs. UVA (5J/cm^2^)-treated controls. ^o^, *p*<0.05 vs. UVA (5J/cm^2^) and aspartic acid (100 μM)-treated controls. The results were verified by repeating the experiments four times, each of which was conducted in duplicate. AA: aspartic acid, dBcAMP: dibutyryl cAMP

**Fig 7:**
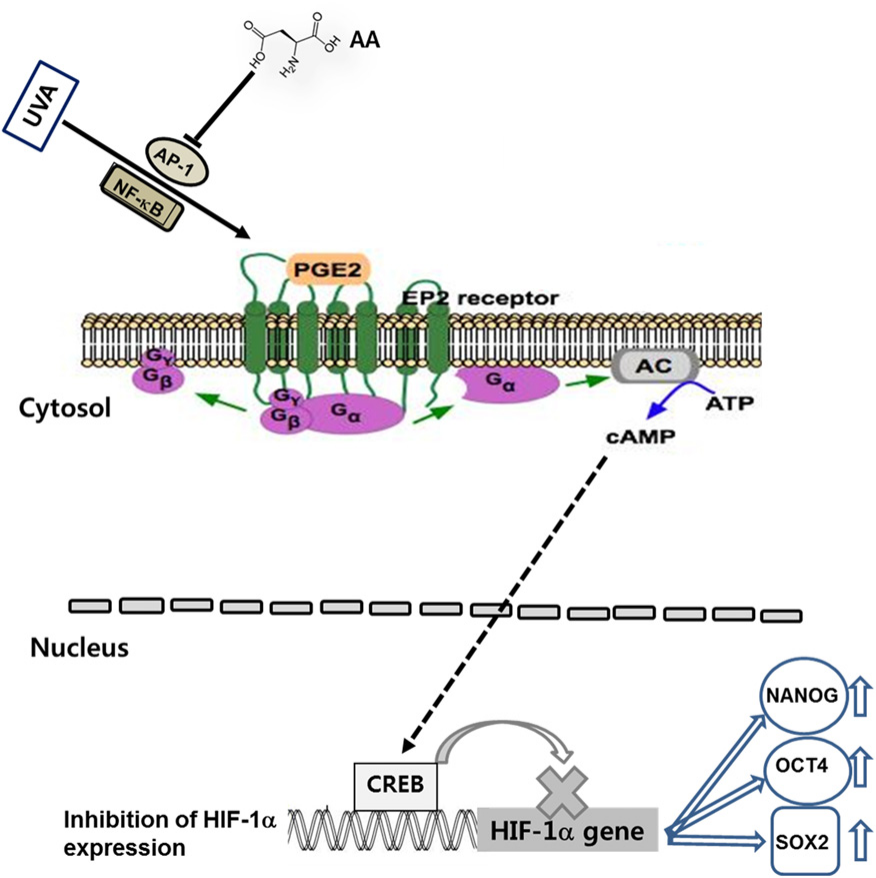
Mechanisms of aspartic acid effects against UVA-induced attenuation of stemness of stem cells. UVA irradiation induces production of PGE_**2**_ and its downstream molecule, cAMP through activation of AP-1 and NF-κB. cAMP molecule sequentially reduces expression of HIF-1α gene through CREB activation, consequently downregulating expression of stemness genes such as NANOG, SOX2, and OCT4. In the UVA irradiation-induced signaling pathway, aspartic acid attenuates UVA-induced effects on expression of stemness genes by inhibiting AP-1, which is upstream of PGE_**2**_ production. AA: aspartic acid

Our previous results suggested that aspartic acid enhances expression of stemness genes by downregulating PGE_2_-cAMP-HIF-1α signaling through activation of AP-1. These results were further confirmed by experiments using siRNA for HIF-1α, PGE_2_, and cAMP. As shown in Fig 6, aspartic acid treatment reduced the effects of UVA on the expression of stemness-related genes. Specifically, the reduced expression of both HIF-1α gene (Fig 6A) and OCT4, NANOG, and SOX2 genes (Fig 6B) caused by UVA irradiation was increased by aspartic acid treatment. However, the effects of aspartic acid were attenuated by treatment of PGE_2_ and cAMP as well as knock-down of the HIF-1α gene. These results indicate that aspartic acid operates upstream of PGE_2_, cAMP and HIF-1α molecules, suggesting that aspartic acid effects are mediated by downregulating PGE_2_-cAMP-HIF-1α signaling through inhibition of AP-1. The action mechanisms of aspartic acid were shown in detail in Fig 7.

## Discussion

It was reported that UVA irradiation suppresses the stemness properties of hAMSCs and its inhibitory mechanisms involve upregulation of PGE_2_ production through the activation of AP-1 and NF-κB [7]. Our studies suggest that the effects of UVA irradiation were found to be attenuated by aspartic acid, which was mediated by reducing the production of PGE_2_ through the inhibition of JNK and p42/44 MAPK, which consequently inhibited PGE_2_-cAMP-HIF-1α signaling and increased the stemness of hAMSCs.

Several papers have reported that the PGE_2_-cAMP signaling pathway downregulates the expression of HIFs [15,16]. In addition, UVA irradiation was reported to downregulate HIF-1α expression through activation of the PGE_2_-cAMP signaling pathway which is mediated by the activation of AP-1 and NF-κB [7]. In this study, after investigating effects of aspartic acid on the PGE_2_-cAMP signaling pathway which is induced by UVA, we found that aspartic acid suppressed UVA-induced production of PGE_2_ as well as cAMP, a downstream molecule of PGE_2_, through the inhibition of JNK and p42/44 MAPK. Collectively, these results indicate that aspartic acid attenuates the effects of UVA by selectively inhibiting activation of JNK and P42/44 MAPK. This finding suggests that aspartic acid may be used as an UVA-antagonizing agent to recover the stemness of hAMSCs.

Stemness genes such as OCT4, NANOG, and SOX2 are crucial transcription factors regulating stem cell self-renewal [17]. Expression of these genes was reported to be reduced by UVA irradiation through the downregulation of HIF-1α, but not of HIF-2α [7]. In a present study, we found that reduced expression levels of OCT4, NANOG, and SOX2 by UVA irradiation were all increased by aspartic acid, suggesting that aspartic acid attenuates the effects of UVA irradiation on hAMSCs by upregulating expression of OCT4, NANOG, and SOX2 genes.

UVA irradiation is an environmental factor which exerts its several effects on cells and tissues. In addition, research has recently focused on adipose tissue due to the increase in the rates of obesity. In addition, the effects of UVA irradiation on adipose tissue, adipocytes, and adipose tissue-derived mesenchymal stem cells have been reported in our previous studies, which demonstrated that UVA irradiation inhibits adipogenic differentiation [3] as well as the stemness of hAMSCs [7]. These reports suggest that UVA irradiation is deleterious to the normal stemness biology of hAMSCs, despite its anti-adipogenic effect, which may be beneficial for reducing diet-induced obesity. In addition, hAMSCs have received a lot of attention as a therapeutic candidate for cell therapy because, compared to other mesenchymal stem cells, hAMSCs are relatively easy to obtain in large quantities. Therefore, although N-methyl-D-aspartate (NMDA), a derivative of aspartic acid, was reported to induce neurotoxicity [18], aspartic acid, which was shown to maximally attenuate the effects of UVA irradiation at the concentration of 100 μM without any cytotoxicity in *in vitro* studies, may be used to restore the UVA-damaged stemness properties of hAMSCs.

Taken together, the results of this study demonstrate that aspartic acid attenuates the effects of UVA irradiation by reducing the production of PGE_2_ through the inhibition of JNK and p42/44 MAPK, consequently recovering the stemness of hAMSCs.

## Supporting Information

S1 Fig The average expression stability value of each gene determined using the NormFinder software. The NormFinder algorithm was used to rank the five irradiated hAMSC reference gene candidates according to their expression stability. As revealed by the analysis, GAPDH, which was characterized by a stability value of 0.128, had the most stable expression levels and thus was selected by the algorithm as the best choice for a single reference gene for expression studies in normal and UVA-irradiated hAMSCs. ACTB and TUBB1 were ranked as the second- and third-best reference genes, respectively. The program additionally identified GAPDH and ACTB as the best combination of two reference genes. GAPDH: glyceraldehydes-3-phosphate dehydrogenase, ACTB: β-actin, TUBA 1A: tubulin- α1a, TUBB1: tubulin-β1, VIM: vimentin.
(TIF)

S1 Table The intragroup variation as determined by the NormFinder software for each gene. Normfinder was also used to calculate the intragroup variation. The variation of the five candidate genes in normal and matched irradiated hAMSC pairs are presented in S1 Table. The variation of GAPDH in the normal and matched irradiated hAMSCs was the lowest, whereas TUBA1A and VIM had the largest variance. GAPDH: glyceraldehydes-3-phosphate dehydrogenase, ACTB: β-actin, TUBA 1A: tubulin- α1a, TUBB1: tubulin-β1, VIM: vimentin.
(TIF)

## References

[1] Situm M, Buljan M, Cavka V, Bulat V, Krolo I, Mihić LL. Skin changes in the elderly people—how strong is the influence of the UV radiation on skin aging? Coll Antropol. 2010;34: 9–13. 21302699

[2] Halliday GM, Rana S. Waveband and dose dependency of sunlight-induced immunomodulation and cellular changes. Photochem Photobiol. 2008;84: 35–46. 10.1111/j.1751-1097.2007.00212.x 18173699

[3] Lee J, Lee J, Jung E, Kim YS, Roh K, Jung KH, et al Ultraviolet A regulates adipogenic differentiation of human adipose tissue-derived mesenchymal stem cells via up-regulation of Kruppel-like factor 2. J Biol Chem. 2010; 285: 32647–32656. 10.1074/jbc.M110.135830 20693579 PMC2952267

[4] Mitani H, Koshiishi I, Toyoda H, Toida T, Imanari T. Alterations of hairless mouse skin exposed to chronic UV irradiation and its prevention by hydrocortisone. Photochem Photobiol. 1999;69: 41–46. 10063799

[5] Sunderkötter C, Kuhn A, Hunzelmann N, Beissert S. Phototherapy: a promising treatment option for skin sclerosis in scleroderma?. Rheumatology (Oxford). 2006;45 Suppl 3: iii52–iii54. 16987838 10.1093/rheumatology/kel293

[6] Horn TD, Morison WL, Farzadegan H, Zmudzka BZ, Beer JZ. Effects of psoralen plus UVA radiation (PUVA) on HIV-1 in human beings: a pilot study. J Am Acad Dermatol. 1994;31: 735–740. 7929918 10.1016/s0190-9622(94)70234-9

[7] Lee J, Jung E, Hyun JW, Park D. Ultraviolet A regulates the stemness of human adipose tissue-derived mesenchymal stem cells through downregulation of the HIF-1α via activation of PGE(2)-cAMP signaling. J Cell Biochem. 2012;113: 3681–3691. 10.1002/jcb.24241 22753248

[8] Boyer LA, Lee TI, Cole MF, Johnstone SE, Levine SS, Zucker JP, et al Core transcriptional regulatory circuitry in human embryonic stem cells. Cell. 2005;122: 947–956. 16153702 10.1016/j.cell.2005.08.020 PMC3006442

[9] Moreno-Manzano V, Rodríguez-Jiménez FJ, Aceña-Bonilla JL, Fustero-Lardíes S, Erceg S, Dopazo J, et al FM19G11, a new hypoxia-inducible factor (HIF) modulator, affects stem cell differentiation status. J Biol Chem. 2010;285: 1333–1342. 10.1074/jbc.M109.008326 19897487 PMC2801260

[10] Forristal CE, Wright KL, Hanley NA, Oreffo RO, Houghton FD. Hypoxia inducible factors regulate pluripotency and proliferation in human embryonic stem cells cultured at reduced oxygen tensions. Reproduction. 2010;139: 85–97. 10.1530/REP-09-0300 19755485 PMC2791494

[11] Rycerz K, Jaworska-Adamu JE. Effects of aspartame metabolites on astrocytes and neurons. Folia Neuropathol. 2013;51: 10–17. 23553132 10.5114/fn.2013.34191

[12] Nadler JV. Aspartate release and signalling in the hippocampus. Neurochem Res. 2011;36: 668–676. 10.1007/s11064-010-0291-3 20953700

[13] Lee J, Kim YS, Park D. Rosmarinic acid induces melanogenesis through protein kinase A activation signaling. Biochem Pharmacol. 2007;74: 960–968. 17651699 10.1016/j.bcp.2007.06.007

[14] Canino C, Mori F, Cambria A, Diamantini A, Germoni S, Alessandrini G, et al SASP mediates chemoresistance and tumor-initiating-activity of mesothelioma cells. Oncogene. 2012;31: 3148–3163. 10.1038/onc.2011.485 22020330

[15] Sakata D, Yao C, Narumiya S. Prostaglandin E2, an immunoactivator. J Pharmacol Sci. 2010;112: 1–5. 20051652 10.1254/jphs.09r03cp

[16] Torii S, Okamura N, Suzuki Y, Ishizawa T, Yasumoto K, Sogawa K. Cyclic AMP represses the hypoxic induction of hypoxia-inducible factors in PC12 cells. J Biochem. 2009;146: 839–844. 10.1093/jb/mvp129 19671538

[17] Covello KL, Kehler J, Yu H, Gordan JD, Arsham AM, Hu CJ, et al HIF-2alpha regulates Oct-4: effects of hypoxia on stem cell function, embryonic development, and tumor growth. Genes Dev. 2006;20: 557–570. 16510872 10.1101/gad.1399906 PMC1410808

[18] Asano D, Nakahara T, Mori A, Sakamoto K, Ishii K. Regression of retinal capillaries following N-methyl-D-aspartate-induced neurotoxicity in the neonatal rat retina. J Neurosci Res. 2015;93: 380–390. 10.1002/jnr.23492 25284371

