# Supplementary figures and images for: Antagonizing Effects of Aspartic Acid against Ultraviolet A-Induced Downregulation of the Stemness of Human Adipose Tissue-Derived Mesenchymal Stem Cells
Source: PLoS One. 2015 Apr 24;10(4):e0124417. doi: 10.1371/journal.pone.0124417 (PMC4409053; doi:10.1371/journal.pone.0124417)

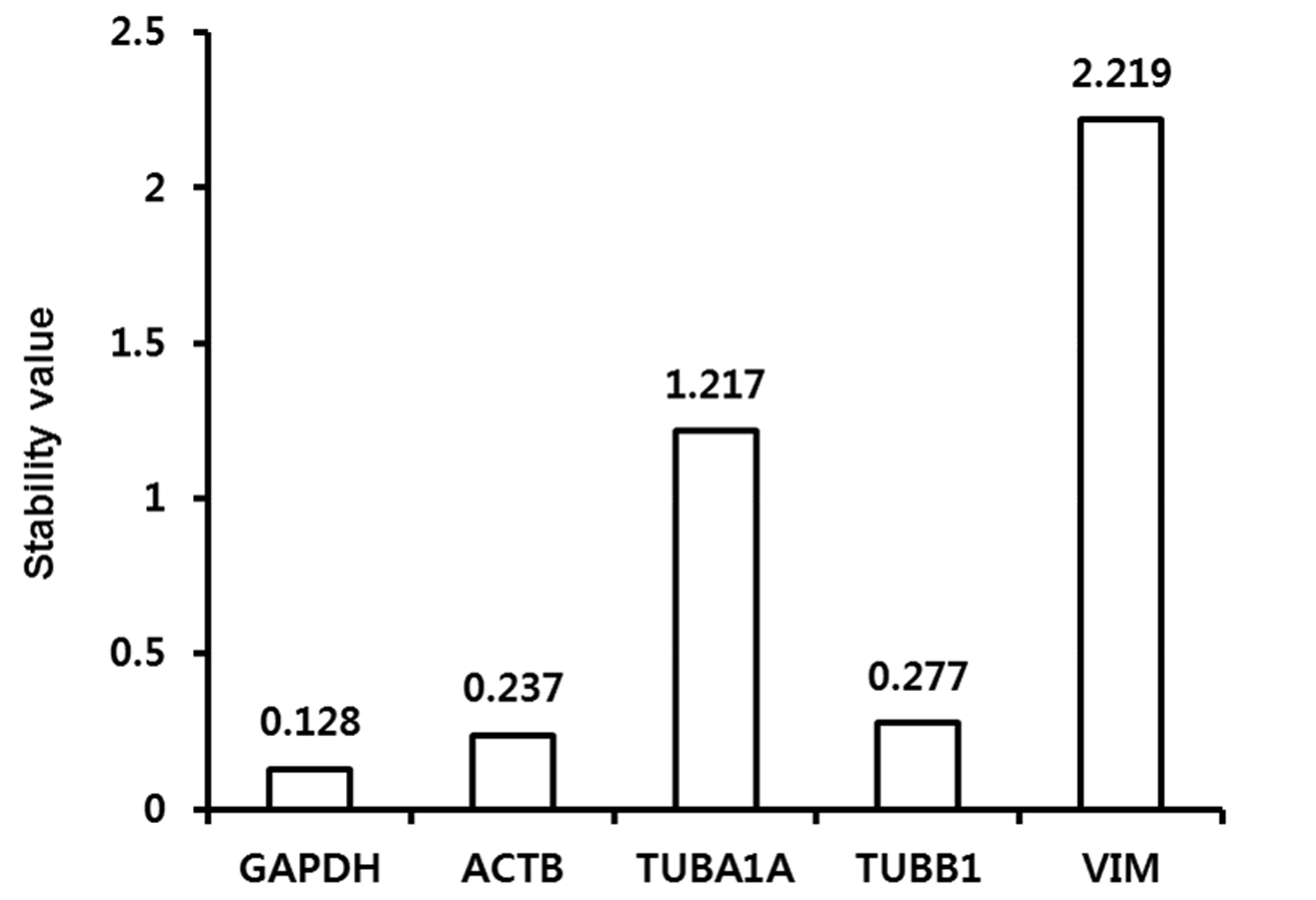

Supplement: S1 Fig — The NormFinder algorithm was used to rank the five irradiated hAMSC reference gene candidates according to their expression stability. As revealed by the analysis, GAPDH, which was characterized by a stability value of 0.128, had the most stable expression levels and thus was selected by the algorithm as the best choice for a single reference gene for expression studies in normal and UVA-irradiated hAMSCs. ACTB and TUBB1 were ranked as the second- and third-best reference genes, respectively. The program additionally identified GAPDH and ACTB as the best combination of two reference genes. GAPDH: glyceraldehydes-3-phosphate dehydrogenase, ACTB: β-actin, TUBA 1A: tubulin- α1a, TUBB1: tubulin-β1, VIM: vimentin. (TIF) [file pone.0124417.s001.tif]

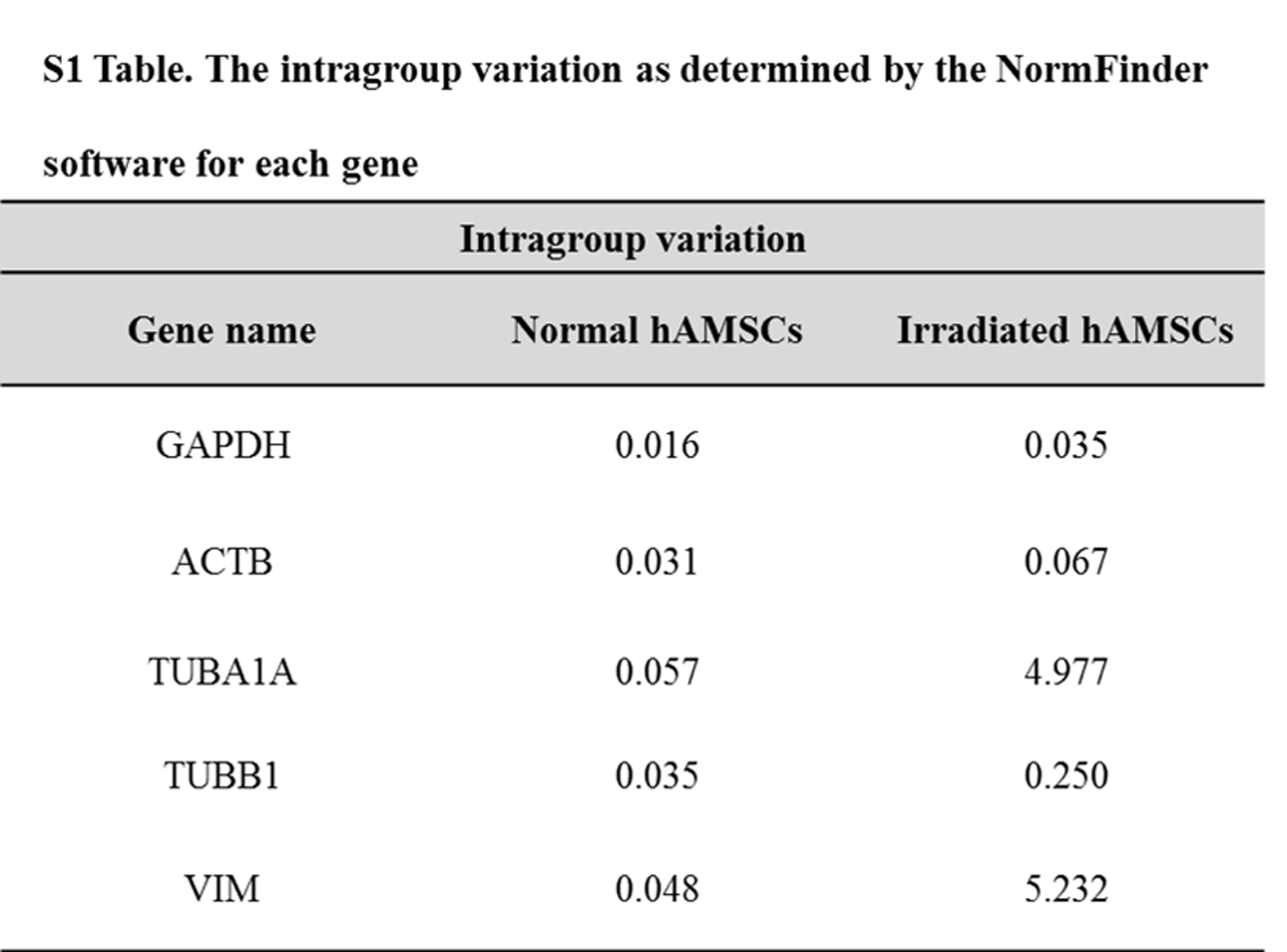

Supplement: S1 Table — Normfinder was also used to calculate the intragroup variation. The variation of the five candidate genes in normal and matched irradiated hAMSC pairs are presented in S1 Table. The variation of GAPDH in the normal and matched irradiated hAMSCs was the lowest, whereas TUBA1A and VIM had the largest variance. GAPDH: glyceraldehydes-3-phosphate dehydrogenase, ACTB: β-actin, TUBA 1A: tubulin- α1a, TUBB1: tubulin-β1, VIM: vimentin. (TIF) [file pone.0124417.s002.tif]
